# A Sketch of Practical Meteorology

**Published:** 1855-07

**Authors:** Sanford B. Hunt


### ART. III.—*A Sketch of Practical Meteorology.*

By SANFORD B. HUNT, M. D.

It has for some time been my intention in presenting my analysis of the meteorological conditions of the last year, to connect with it a brief statement of some of the difficulties which obstruct this path of research, and to give a practical sketch of the manipulations, calculations, and formulæ in use. In so doing I shall present a partial apology for the errors and inconsistencies which have crept into my tables, and an explanation of a change in the manner of reducing observations, which will commence with June, and change the comparative value of last year's observations.

I shall not stop to discuss the probable value af such observations, further than to remark that they are evidently underestimated. A distinguished meteorologist lately said to me that he was much mortified to read the complimentary notices bestowed upon him by the scientific press. In almost every instance the style of diction and frequent errors of fact, made it very evident that the learned writers, though perhaps capable of appreciating results, were remarkably ignorant of the first principles of the science they were discussing.

Such was the case, to a large extent, in the case of the New Orleans Sanitary Report. The comments of the medical press upon Dr. Barton's labors, though kind and flattering, were not, as a general thing, intelligent. The only instance of adverse criticism—the furious tirade of the New Orleans Medical Journal—though intended for a laborious and analytical criticism, displayed an amount of ignorance of the subject which it must have been the work of years to accumulate.

The fact that watery vapor is an almost constant but exceedingly changeable constituent of the atmosphere, coupled with the well known effects of extreme dryness, or high saturation as in the vapor bath, lead at once to the idea that the element which at 120° F. will produce rapid, and if continued, fatal syncope, must, even at temperatures much lower, produce a corresponding but lessened effect upon the organism. Add to this the agency of heat and moisture in developing the fermentative process—zymosis—in intensifying, dissolving, and conveying to the circulation the elements of malaria, and it is evident that the subject is one worthy of study and fruitful of results, that when the habits and causes of high humidity are understood, the means of avoiding them, and with them epidemic disease, will readily suggest themselves, and thus achieve another of those noblest triumphs of medical learning, the prevention rather than the cure of disease.

A portion of the philosophy of atmospheric humidity is obscure. The pressure of vapor on columns of mercury at certain temperatures, has been ascertained, and from observations made at high temperatures, a scale of weights of aqueous vapor has been calculated, which assigns to each temperature a certain capacity for moisture. Thus a cubic foot of vapor at a dew-point ("point of maximum density") of 95°, weighs 17.009 grs., which, at 32° the same cubic foot would weigh but 2.539 grs., the surplus having been precipitated by the cold. There is a degree of uncertainty about this, the tables having been constructed on the principle that aqueous vapor followed certain mathematical rules of increase of pressure. This is true with high temperatures, but with low temperatures it is certainly not proven to be correct, and the subject requires revision.

These tables are, however, in daily use, from the necessity of the case, and, as undoubtedly *some* direct ratio exists, these figures, if they do not express the fact, may be very valuable for comparative purposes.

Without stopping to discuss the various hygrometers in use, I shall give, briefly, a description of Mason's Hygrometer, the principles on which it is constructed, and the method of its manipulation, in hopes that many readers of this article may be induced to procure it, and to make careful observations of its changes with reference to health and disease.

The instrument consists of two thermometers hung upon the same scale.

A glass fountain between them keeps the bulb of one constantly moistened (by means of a bit of fine gauze) with distilled water. They may be procured from Messrs. McAllister & Bro's, of Philadelphia, at the price of $3.

The dry bulb thermometer gives the temperature of the air—the wet bulb that of evaporation. If the atmosphere be saturated with moisture, it is, of course, incapable of further absorption, and no evaporation takes place. But as the dryness of the atmosphere increases, evaporation becomes more rapid, occasioning a diminution of temperature in the wet bulb. Now as there is a constant ratio between the difference of the two thermometers, and the capacity of the air for moisture, it will be seen that these two temperatures of the air and of evaporation, are the only conditions necessary for observation.

The dew-point is reached from them by mathematical formulæ. It has been the custom of nearly all meteorologists to obtain it, and express it as a necessary part of their tables. This is rather a scientific nicety than a necessity. The simple reading of the two thermometers expresses very nearly all that can be told of the relative amount of vapor. The advantages resulting from deducing the dew-point are, that it gives us, by the aid of the tables furnished with the hygrometer, the exact amount of vapor then existing in a cubic foot of air, and enables us to express the relative humidity in fractional figures. Thus the temperature being 60°, that of evaporation being 58°, the dew-point will be 56.4°. This means simply that the air contains as much moisture as would saturate it at 56.4°. If we wish to express the *relative* amount of moisture, the amount it does contain compared to that which it might contain at 60°, we have but to divide the number of grs. weight at 56.4° by that at 60°, and the process gives us in fractions of one thousand, 896 as the relative humidity—that is the air is $\frac{896}{1000}$ saturated. For uniformity and convenience, if not for their intrinsic value, it is best to follow the general course and make the calculations necessary for ascertaining the dew-point and the relative humidity.

Various formulæ have been proposed. The most accurate of these is that of Regnault, but it is too abstruse for every day use. The formula in use by Prof. Kirkpatrick, of Philadelphia, and furnished with the instrument, is as follows: Multiply the difference between the two thermometers by two, and add one-third the difference. Deduct the sum from the temperature.

E. g., temperature of air 60°, of evaporation 57°. The difference $\times$ by $2 = 6 + \frac{1}{3}$ the difference, $= 7$. $60° - 7 =$ equals 53°, dew-point.

This is a neat and convenient formula, so far as these qualities are concerned. Where it originated, and how correct it may be, I do not know, but it differs very widely in its results from Regnault's formula. It is probably too large in its factors, and makes the difference between dew-point and evaporation, far too great. Nevertheless, for the want of a convenient and easy working formula, I have calculated all my tables up to June, 1855, by it.

Another method, and the one which will hereafter be pursued in the tables given in this Journal, is that used by Dr. Barton, and first proposed by the American Philosophical Society. A comparison of over one hundred calculations made by it with the same made by Regnault's formula, shows but a very trifling difference between the two, and gives me great confidence in its accuracy.

The difference between the two thermometers is multiplied by 105, the sum divided by the wet bulb temperature, and the quotient subtracted from the dry bulb.

E. g., the temperature of the air is 80°, that of evaporation 75°. $105 \times 5 = 525 \div$ by $75 = 7$. $80 - 7 = 73$, the dew-point.

The relative humidity is, of course, ascertained in the usual method. Though this calculation is somewhat laborious to make three times daily, most of the labor can be saved by regularly tabulating each result, and thus no calculation need be made the second time. The saving of labor, in a matter of this kind, is of very great importance.

Next to the hygrometer the barometer is an instrument of importance. The great differences between the readings of different instruments impair the value of compared observations, but the results of a single barometer may be advantageously compared with themselves. The aneroid barometer is a new and very beautiful instrument, constructed on the principle of the different pressures on a vacuum. A brass box is exhausted of air, and by means of delicate adjustments the different pressures of the atmosphere are marked on a dial. That ordinarily in use is the mercurial barometer. It is difficult to adjust to any fixed standard, but it gives comparative pressures well enough. Certain corrections are applied to the mercurial barometer for capillarity and for force of vapor. These are not necessary in ordinary observations, though needed in accurate observation, of heights.

The cheapest *rain-guage* is furnished by Messrs. McAllister. It consists of a funnel of a given diameter, to which is adapted a graduated glass which gives, in fractions of one-thousandth of an inch, the vertical depth of rain.

With these three instruments a very complete set of observations may be made. The barometer should be hung in a hall away from danger of accident. The hygrometer should be placed in a shaded position, in free out door air, away from direct or reflected heat. The latter especially is a source of very common error. The wet bulb should be constantly wetted by the fountain, or if found dry, a little time should elapse after wetting, and the instrument swung to and fro in the air before recording it. Observations should be made at 7, A. M., 2, P. M., and 9, P. M., as an average of those hours gives a fair mean of the whole twenty-four. The record should be made at the time, in ink; and generally, it is best to carry out the dew-point and humidity. Inasmuch as air in motion precipitates moisture more readily than when still, the observation of the hygrometer should be made in free air. Dr. Barton blows with a bellows for a few moments upon the wet bulb. But this should only be done in windy days, or in imitation of the motion of the air at the time. The water used for the fountain should be distilled, or that which has been boiled. The fountain once filled will supply the bulb for several weeks. The cloth or guaze upon the bulb should be changed as often as it becomes dirty. In the winter the fountain is liable to burst from the freezing of its contents. It should, therefore, be removed, and the bulb coated over with a thin film of ice, which will usually last a day or two.

In observing the mercurial barometer, it should be recollected that the cohesion of the mercury to the sides of the tube is considerable, and the instrument should be jarred or shaken before the observation.

The rain-guage should be placed on a post a few feet from the ground, and in a favorable situation for getting a fair average of the water falling. The graduate is too small to hold more than the water of a very light shower —one-tenth of an inch—and accordingly a bottle should be used as a reservoir, and the water of each rain measured from it. To measure the amount of snow falling some precautions are necessary. My method was to leave a dry goods box in the back yard, in such a situation as to be sheltered from direct winds, and to be out of the way of drifts. The box being placed bottom up, the snow accumulating upon it is assumed as the average depth. The rain-guage, inverted, is pressed down upon it. By this means, even if the snow be very deep, the guage packs it beneath itself, forming a solid perpendicular column of snow of the diameter of the guage. The rest of the snow is then carefully swept off, and that beneath the guage is melted and measured as so much water. The box being cleanly swept is ready for another fall of snow.

It only remains to mention the observation of wind and cloud.

The scale used by the Smithsonian Institution is given on the next page and explains itself.

### SCALE OF WINDS.

| Number given by Observer. | Miles per Hour. | Force in lbs. on sq. ft. | Technical Description. |
|---|---|---|---|
| 1 | 2 | 020 | Very light breeze. |
| 2 | 4 | 030 | Gentle breeze. |
| 3 | 12 | 750 | Fresh breeze. |
| 4 | 25 | 3.000 | Strong wind. |
| 5 | 35 | 6.000 | High wind. |
| 6 | 45 | 10.000 | Gale. |
| 7 | 60 | | Strong gale. |
| 8 | 75 | | Violent gale. |
| 9 | 90 | | Hurricane. |
| 10 | 100 | | Most violent hurricane. |

For the exact use of this scale an anemometer is necessary, an instrument which may be constructed on the principle of the spring balance, to act horizontally, with a rachet attached by which the spring is held at the highest tension attained. The instrument may be easily constructed, but most observers will be satisfied with a shrewd guess, and a little practice is sufficient to secure quite uniform results. The direction should also be registered. For instance it is blowing a gale from the west, the record would be made as follows: "6. W."

The record of the wind is important with a view to ascertaining which are the rain-bearing winds, and which have the highest hygrometric condition.

Clouds are reached by the amount of sky covered. Thus the figure 7 represents the sky seven-tenths covered. The character of cloud is expressed by abbreviationst of, he generic names of clouds. Ni., for nimbus, or rain cloud; Cu., for cumulus, or the round, piled-up cloud, so common in the summer; St., for stratus, the horizontal cloud; and Ci., for cirrus, the light, broken, fleecy cloud of the upper air. Combinations of these primitive forms are expressed thus: Ci.-st., for cirus-stratus; Ci.-cu., for cirro-cumulus, &c.

Possibly the detail of this sketch may seem prosy, and, to the initiated, too plain to need description. But I do not know where the same information is to be found in printed form; and if this article should prevent that annoyance and embarrassment on the part of the beginner from which the writer has suffered, for want of precise and tangible knowledge, it will have attained its end.

### TABLE OF CLIMATIC CONDITIONS, AT BUFFALO, FOR THE YEAR ENDING JUNE 1, 1855.

| Months. | Barometer. | In. of Rain. | Temp'ture. | Dew-Point. | Humidity | Remarks. |
|---|---|---|---|---|---|---|
| June,....... | 29. | | 71.39 | 62.53 | .757 | Only the 2, P. M. observations are used, as the 7, A. M., and 2, P. M. observations were not made during the first four months. |
| July, ....... | 29.20 | | 79.67 | 68.30 | .744 | |
| August,..... | 29.20 | | 75.61 | 66.50 | .754 | |
| September,- | 29.20 | *6.000 | 71.47 | 63.83 | .782 | |
| October, .... | 29.16 | 2.010 | 59.84 | 49.63 | .729 | |
| November,. | 28.90 | 7.064 | 42.10 | 33.76 | .781 | |
| December,. | 28.90 | 2.893 | 30.23 | 26.29 | .887 | |
| January,.... | 29.08 | †5.277 | 32.13 | 28.05 | .881 | |
| February,.. | 29.10 | 3.860 | 23.39 | 20.01 | .914 | 6th Feb'y coldest day, 20° below. |
| March,...... | 28.90 | 2.107 | 37.07 | 29.30 | .782 | |
| April. ...... | 29.07 | 2.727 | 49.00 | 38.20 | .725 | |
| May, ....... | 29.03 | 1.865 | 62.93 | 47.80 | .672 | |
| M'ly Mean, | 29.06 | 2.817 | 52.90 | 44.52 | .783 | |

* Estimated at six inches for the four months.

† Two inches added for estimated loss. Rather too small than too large.

### NUMBER OF DAYS IN EACH MONTH ON WHICH IT BLEW.

| Months. | Calm. | Very light breeze. | Gentle breeze. | Fresh breeze. | Strong wind. | High wind. | Gale. | Great gale. | No. of days observ'd | Hour of observation |
|---|---|---|---|---|---|---|---|---|---|---|
| June,....... | 8 | 4 | 9 | 1 | 2 | 1 | | | 25 | 2, P. M. |
| July, ....... | 1 | 18 | 9 | 2 | 1 | | | | 31 | " " " |
| August,..... | 3 | 8 | 9 | 5 | 3 | 2 | | | 31 | " " " |
| September,. | | 20 | 6 | 4 | | | | | 30 | " " " |
| October, .... | 3 | 18 | 4 | 5 | 1 | | | | 31 | All hours. |
| November,. | | 9 | 8 | 4 | 2 | 5 | 2 | | 30 | " " |
| December, . | | 7 | 6 | 9 | 6 | 2 | | 1 | 31 | " " |
| January,.... | | 4 | 5 | 7 | 11 | 1 | 2 | 1 | 31 | " " |
| February,.. | | 9 | 8 | 3 | 3 | 5 | | | 28 | " " |
| March,...... | | 3 | 6 | 5 | 7 | 3 | 1 | | 25 | " " |
| April,....... | 1 | 7 | 7 | 10 | 5 | | | | 30 | " " |
| May, ....... | | 10 | 6 | 2 | | | | | 20 | " " |
| For the year, | 16 | 117 | 83 | 57 | 40 | 19 | 5 | 2 | 343 | |

### DIRECTION OF WINDS.

NUMBER OF DAYS IN EACH MONTH ON WHICH THE WIND BLEW FROM

| Months. | N. | N.E. | E. | S.E. | S. | S.W. | W. | N.W. | Calm | No. of days observ'd | Hours of observation. |
|---|---|---|---|---|---|---|---|---|---|---|---|
| June, ....... | 2 | | | | | 15 | | | 8 | 25 | 2, P. M. |
| July, ....... | | 1 | 1 | 1 | | 22 | 4 | 2 | | 31 | " " " |
| August, ..... | 1 | 2 | | | 1 | 14 | 7 | 2 | 3 | 30 | " " " |
| September, .. | 5 | 1 | | | 2 | 15 | 4 | 3 | | 30 | " " " |
| October, .... | 1 | 1 | | 2 | 8 | 8 | 3 | 5 | 3 | 31 | " " " |
| November, .. | | 1 | 2 | 1 | 7 | 9 | 7 | 2 | | 28 | " " " |
| December, . | 3 | 1 | 1 | | 5 | 10 | 3 | 7 | | 31 | " " " |
| January, .... | 1 | 7 | | 4 | 2 | 8 | 4 | 4 | | 31 | " " " |
| February, .. | | 3 | 1 | | 2 | 5 | 16 | 1 | 1 | 28 | " " " |
| March, ...... | 1 | | | | 8 | 12 | 3 | 1 | | 25 | " " " |
| April, ...... | | | | 1 | 5 | 10 | 11 | | 2 | 29 | " " " |
| May, ....... | 2 | 1 | 1 | | 2 | 9 | 3 | 1 | | 19 | " " " |
| For the year, | 16 | 18 | 6 | 9 | 42 | 135 | 65 | 28 | 17 | 338 | |

### AMOUNT OF CLOUD.

NUMBER OF DAYS IN EACH MONTH ON WHICH THE SKY WAS 10THS COVERED.

| Months. | 0 | 1/10 | 2/10 | 3/10 | 4/10 | 5/10 | 6/10 | 7/10 | 8/10 | 9/10 | 10/10 | Fog or Haze. | Days observ'd | Rained |
|---|---|---|---|---|---|---|---|---|---|---|---|---|---|---|
| June, ....... | 7 | 2 | | | | 6 | | | 3 | 1 | | 2 | 19 | 7 days. |
| July, ....... | 12 | 7 | 3 | 5 | 1 | 2 | | 1 | | | | | 31 | 6 " |
| August, ..... | 4 | 1 | 4 | 4 | | 5 | | 1 | | | 1 | 8 | 30 | 5 " |
| September, .. | 9 | 3 | 6 | 1 | 1 | 3 | 1 | | 1 | 2 | 3 | | 30 | 5 " |
| October, .... | 8 | 1 | 2 | 2 | 2 | 3 | 1 | | 2 | 1 | 10 | | 30 | 10 " |
| November, . | 3 | 2 | 1 | | | 2 | | 1 | | 3 | 18 | | 30 | Snow 7. Rain 9. |
| December, . | 3 | | | 1 | 3 | 4 | 1 | 2 | 1 | 2 | 14 | | 31 | Snow 8 days. |
| January, .... | 5 | 1 | | | | 1 | | | 2 | 3 | 14 | | 26 | Snow 11. Rain 1. |
| February, .. | 1 | | | 3 | | | 1 | | 1 | | 22 | | 28 | Snow 3 days. |
| March, ...... | 2 | 1 | 2 | | | 2 | | | 1 | | 17 | | 25 | Snow 4. Rain 4. |
| April, ...... | 13 | 1 | | | | 3 | 1 | | 1 | | 10 | 1 | 29 | Rain 6. |
| May, ....... | 7 | | 1 | 1 | | 5 | | | | 1 | 4 | | 19 | Snow 1. Rain 3. |
| | 74 | 19 | 19 | 17 | 7 | 39 | 5 | 5 | 12 | 13 | 113 | 11 | 328 | 90 days. |

